# Persistence of a pKPN3-Like CTX-M-15-Encoding IncFII_K_ Plasmid in a *Klebsiella pneumonia* ST17 Host during Two Years of Intestinal Colonization

**DOI:** 10.1371/journal.pone.0116516

**Published:** 2015-03-04

**Authors:** Iren Høyland Löhr, Nils Hülter, Eva Bernhoff, Pål Jarle Johnsen, Arnfinn Sundsfjord, Umaer Naseer

**Affiliations:** 1 Department of Medical Microbiology, Stavanger University Hospital, Stavanger, Norway; 2 Department of Clinical Medicine, University of Bergen, Bergen, Norway; 3 Department of Pharmacy, UiT the Arctic University of Norway, Tromsø, Norway; 4 Department of Medical Biology, UiT the Arctic University of Norway, Tromsø, Norway; 5 Department of Microbiology and Infection Control, University Hospital of North Norway, Tromsø, Norway; 6 Department of Food-borne Infections, Norwegian Institute of Public Health, Oslo, Norway; Université d'Auvergne Clermont 1, FRANCE

## Abstract

**Objectives:**

To characterize the CTX-M-15-encoding plasmid in a *Klebsiella pneumoniae* ST17 strain, responsible for an outbreak at a Norwegian neonatal intensive care unit and subsequent colonization of affected children for up to two years. To identify plasmid-mediated features relevant for the outbreak dynamics, and to investigate the plasmids capability of horizontal transfer, its segregational stability and plasmid-mediated fitness costs.

**Methods:**

Plasmid profiling was performed by *S1*-nuclease PFGE, PCR-based replicon typing and Southern blot-hybridization. The complete sequence of the CTX-M-15-encoding plasmid was obtained by 454 sequencing. Plasmid self-transferability was investigated by broth- and filter mating, segregational stability was explored by serial passage, and plasmid-conferred fitness costs were examined in pairwise head-to-head competitions and by growth rate comparisons.

**Results:**

CTX-M-15 was encoded by a ~180 kb IncFII_K_ plasmid in *K. pneumoniae* ST17. *S1*-nuclease PFGE profiles of the first and the last CTX-M-15-producing *K. pneumoniae* isolates, recovered from the four children colonized the longest, suggested that the plasmid was stably maintained during intestinal carriage of up to two years. The DNA sequence of the pKPN3-like plasmid, pKp848CTX, uncovered a Tn*3*-like antibiotic resistance region and multiple heavy metal- and thermoresistance determinants. Plasmid pKp848CTX could not be transferred to *Escherichia coli in vitro* and we found no evidence to support horizontal plasmid transfer *in vivo*. Segregational plasmid loss ranging from 0.83% to 17.5% was demonstrated in evolved populations *in vitro*, but only minor fitness costs were associated with plasmid-carriage.

**Conclusions:**

Plasmid pKp848CTX encodes phenotypic traits, which may have had an impact on the fitness and survival of the *K*. *pneumoniae* ST17 strain in the outbreak setting. The antibiotic resistance plasmid pKp848CTX was stably maintained during two years of intestinal colonization, conferring negligible fitness cost to its host, and thus seem well adapted to its *K. pneumoniae* host.

## Introduction

CTX-M type extended-spectrum beta-lactamases (ESBLs) are the most common ESBLs in clinical relevant Enterobacteriaceae worldwide, challenging antibiotic treatment of community and hospital acquired infections [1–3]. Both the dissemination of successful clones, such as *Escherichia coli* ST131, and the horizontal spread of epidemic plasmids encoding CTX-M have contributed to the present situation [4–7].

Genes encoding the most commonly encountered CTX-M type ESBL, CTX-M-15, are most often located on plasmids belonging to the incompatibility family IncF, but may also be encoded by plasmids of other Inc types, such as IncR,-I1,-A/C,-L/M and-N [8–11]. The IncF family consists of narrow-host range low copy number plasmids, which vary in size from 50 to 200 kb. Their host range is limited to Enterobacteriaceae, where they are commonly well adapted [12]. IncF replicons may be classified into IncFIA,-FIB,-FIC and-FII. Based on sequence variations and host preference, the FII replicon has been further subdivided into FII_S_ (*Salmonella* spp.), FII_Y_ (*Yersinia* spp.) and FII_K_ (*Klebsiella* spp.) [13]. In *K*. *pneumoniae*, IncFII_K_ plasmids have been associated with several clinical important beta-lactamases, including CTX-M-15, KPC-2, KPC-3 and NDM-1 [10,14–19]. IncFII_K_ plasmids encoding CTX-M-15 have also been linked to diverse *K*. *pneumoniae* outbreak strains [20–23].

Traditionally, plasmids were considered to confer a biological burden to their host [24]. However, studies have demonstrated that costs associated with plasmid carriage may be ameliorated, or plasmid-carriage may even improve the host’s fitness, through compensatory mutations during co-evolution of host and plasmid [24–26]. Starikova *et al*. recently showed that initial high biological costs associated with the acquisition of a plasmid encoding antibiotic resistance was quickly compensated for, even in the absence of a selective antibiotic pressure [27]. However, thus far, plasmid stability and plasmid-conferred fitness costs have not been investigated during long-term colonization in a human host.

In this study, we investigate the characteristics of a CTX-M-15-encoding plasmid when hosted by a multidrug resistant *K*. *pneumoniae* ST17 strain (CTX-M-15-Kp). CTX-M-15-Kp caused an outbreak in a Norwegian neonatal intensive care unit (NICU) in 2008–09 colonizing 56 children [28], in 49 of whom, persistent intestinal carriage was documented for up to two years in a follow-up study [29]. In the present study, we determine the full DNA sequence of the CTX-M-15-encoding plasmid, and inspect features relevant for the dynamics of the NICU-outbreak including survival and spread in the NICU environment. We furthermore investigate the maintenance of the plasmid in CTX-M-15-Kp in the four children colonized the longest (18–24 months). With respect to the dynamics between the plasmid and its bacterial host, we found no evidence of conjugational self-transfer of the plasmid to other Enterobacteriaceae during human intestinal colonization, or in laboratory experiments. The plasmid seemed to persist in its host strain during two years of colonization. However, segregational plasmid loss appeared frequently *in vitro* in evolving populations, although plasmid-carriage conferred low or no biological cost to its host.

## Materials and Methods

### Bacterial isolates

Ten CTX-M-15-Kp isolates recovered during the NICU outbreak and the follow-up study were selected for plasmid analyses, including the first and the last faecal isolate recovered from child 1–4, who were colonized the longest (18–24 months), the first isolate recovered from child 5, that was the only child from whom a CTX-M-15-producing strain other than CTX-M-15-Kp was detected during follow-up of 49 children, and a breast milk isolate recovered from the mother of child 1 during the outbreak investigations, which was considered the index isolate [28] (Table 1). All CTX-M-15-Kp isolates were indistinguishable by *Xba*I-PFGE in previous studies [28,29]. We furthermore included one CTX-M-15-producing *E*. *coli* isolate, recovered from a follow-up sample from child 5 (Table 1).

**Table 1:** Characteristics and plasmid profiles of selected CTX-M-15-Kp (Kp) and CTX-M-15-producing *E*. *coli* (Ec) isolates.

Subject	Months colonized	Isolate			PFGE-type^a^	Co-resistance^b^	Inc type^c^	Plasmid size (kb)^d^	Southern blot-hybridization		
		Sample	Source	ID					*bla*_CTX-M_	IncFII_K_	IncR
Mother child 1		Index	BM	Kp2177	1	GEN, STX	FII_K_, R	~70, ~180	~180	~180	~70
Child 1	18	First	F	Kp2158	1	GEN, STX	FII_K_, R	~70, ~180	~180	~180	~70
		Last	F	Kp734	1	GEN, STX	FII_K_, R	~70, ~180	~180	~180	~70
Child 2	19	First	F	Kp2180	1	GEN, STX	FII_K_, R	~70, ~180	~180	~180	~70
		Last	F	Kp723	1	GEN, STX	FII_K_, R	~70, ~180	~180	~180	~70
Child 3	22	First	F	Kp2223	1	GEN, STX	FII_K_, R	~70, ~180	~180	~180	~70
		Last	F	Kp798	1	GEN, STX	FII_K_, R	~70, ~180	~180	~180	~70
Child 4	24	First	F	Kp2166	1	GEN, STX	FII_K_, R	~70, ~180	~180	~180	~70
		Last	F	Kp848	1	GEN	FII_K_	~180	~180	~180	-
Child 5	9	First	F	Kp2189	1	GEN, STX	FII_K_, R	~70, ~180	~180	nd	nd
			F	Ec567	nd	-	I1, FIA	~10, ~20, ~40, ~75	~75	nd	nd

(BM) Breast milk; (F) Faeces; (GEN) gentamicin; (SXT) trimethoprim/sulfamethoxazole; (nd) not determined

^a^PFGE of *XbaI*-digested DNA.

^b^Gradient test (Liofilchem).

^c^PCR-based replicon typing.

^d^PFGE of *S1*-nuclease digested DNA.

### Ethics statement

The study was approved by the Regional Committee for Medical and Health Research Ethics, Western Norway (Reference: 096.09). Written statements of informed consent were obtained from the parents of the children.

### Plasmid typing and profiling

Plasmid replicons of CTX-M-15-producing isolates were detected and typed by PCR-based replicon typing (PBRT) as proposed [13,30] using the PBRT kit from DIATHEVA (DIATHEVA, Fano PU, Italy) according to the manufacturer’s instructions. Plasmid profiling was performed by PFGE of *S1*-nuclease (Promega, Madison, WI, USA) digested total DNA [31,32]. Briefly, PFGE was run in a Chef-DR III System (Bio Rad, Oslo, Norway) at 14°C, with pulse time 1–20 s, at 6 V/cm on a 120° angle in 0.5xTBE buffer for 15 h. The Low Range ladder (New England BioLabs, UK) was used as plasmid size marker. Each band on the gel was considered a linearized plasmid. Plasmid DNA bands were transferred from *S1*-nuclease PFGE gels to positively charged nylon membranes using the Vacuum Blotter Model 785 (Bio Rad). CTX-M-15-encoding plasmids were identified through Southern blot-hybridization with *bla*_CTX-M_ specific probes. The replicon types of both CTX-M-15-encoding plasmids and additional plasmids were determined through hybridization with replicon specific probes. The DIG High Prime DNA Labelling and Detection Starter Kit I (Roche Diagnostics, Mannheim, Germany) was used for labelling and detection of PCR-generated probes. The primers used for the generation of *bla*_CTX-M_, IncR and IncFII_K_ specific probes have been described elsewhere [13,33,34] and are listed in Table 2.

**Table 2:** Primers used in this study.

Name	DNA sequence	Amplicon size (bp)	Reference
*bla*_CTX-M_ FW	5’-SCS ATG TGC AGY ACC AGT AA-3’	862	[33]
*bla*_CTX-M_ RW	5’-ACC AGA AYV AGC GGB GC-3’		
IncFII_K_ FW	5’-TCTTCTTCAATCTTGGCGGA-3’	142–148^a^	[13]
IncFII_K_ RW	5’-GCTTATGTTGCACRGAAGGA-3’		
IncR FW	5’-TCG CTT CAT TCC TGC TTC AGC-3’	251	[34]
IncR RW	5’-GTG TGC TGT GGT TAT GCC TCA-3’		

^a^Variable amplicon sizes may be obtained for this replicon.

### Plasmid sequencing and bioinformatics

Plasmid DNA was purified from Kp848 (the last follow-up isolate from child 4 containing only the CTX-M-15-encoding plasmid, Table 1) using the Qiagen large construct kit (Qiagen). A shotgun library was constructed and single-end pyrosequencing reads were obtained applying the 454 Genome Sequencer FLX-system (Roche Diagnostics, Indianapolis, IN, USA). Sequencing was performed by Eurofins MWG Operon (Ebersberg, Germany). Reads were assembled *de novo* using the Newbler software (Roche Diagnostics). Gap closure was performed by sequence-based bridging. Artemis version 15 (Welcome trust Sanger Institute, Hinxton, UK) was used for alignment and annotation. Plasmid sequence comparison with previously published plasmids was performed in WebAct and ACT version 12 (Welcome Trust Sanger Institute). Plasmid MLST (pMLST) was performed using the pMLST version 1.2 of the Center for Genomic Epidemiology (Lyngby, Denmark) [35] as proposed [13,36].

### Plasmid transfer

***In vivo*:** To detect possible self-transfer events of the CTX-M-15-encoding plasmid from CTX-M-15-Kp to other Enterobacteriaceae strains during intestinal colonization, all CTX-M-15-producing isolates other than CTX-M-15-Kp recovered during follow-up of 49 children (n = 1) were characterized by PBRT, *S1*-nuclease PFGE and Southern blot-hybridization as described above. Screening for ESBL-producing Enterobacteiaceae and detection of *bla*_CTX-M-15_ by PCR and DNA sequencing was performed as previously described [29,31,37].

***In vitro*:** Transferability of the CTX-M-15-encoding plasmid from selected CTX-M-15-Kp isolates (the first and the last isolate from child 1–4) to a rifampicin resistant plasmid-free recipient strain, *E*. *coli* J53–2, was investigated by broth mating under the following conditions: strains were cultured overnight (ON) in LB broth at 35°C with slight agitation. ON cultures were diluted 1:100 and incubated at 35°C with slight agitation until reaching the logarithmic growth phase (measured by OD_600nm_ of ~0.5). Donor and recipient cultures were mixed 1:9 and 1:1, incubated at 30°C and 35°C in plain LB broth and in LB broth supplemented with ciprofloxacin (0.08 mg/L), with careful shaking for 4 h and ON (i.e. 16 h). Aliquots of 100 μL mixed culture (diluted 10^-3^ to 10^-6^) were plated on LB plates containing cefotaxime (2 mg/L) and rifampicin (100 mg/L). The clinical *K*. *pneumoniae* strain, Kp2200 (ST 485), containing a self-transferable IncI1 CTX-M-15-encoding plasmid was used as a positive control for the experimental setup.

Furthermore, re-transferability of the CTX-M-15-encoding plasmid was tested by filter mating between CTX-M-15-Kp and corresponding rifampicin resistant plasmid-free segregant isolates (i.e. CTX-M-15-Kp isolates that had lost the CTX-M-15-encoding plasmid during serial passage, see below) under the following conditions: both donor and recipient strains were cultured ON in LB broth at 37°C with slight agitation. ON cultures were diluted 1:1000, mixed 1:1 and spotted in 40 μL droplets on plain LB plates. After 8 h of growth at 37°C, cell lawns were scraped off, re-suspended in PBS and plated on LB plates supplemented with cefotaxime (8 mg/L) and rifampicin (100 mg/L) and incubated ON at 37°C.

### Plasmid stability

***In vivo*:** To detect CTX-M-15-Kp isolates, that possibly had lost *bla*_CTX-M-15_ or the CTX-M-15-encoding plasmid during intestinal colonization, the two first ESBL-negative faecal follow-up samples from 35/49 children (follow-up samples were obtained monthly during the first year of follow-up, thereafter every three months) were screened for ESBL-negative isogenic *K*. *pneumoniae* strains (i.e. *in vivo* segregants devoid of the CTX-M-15 phenotype). Frozen ESBL-negative samples were inoculated on modified McConkey agar and incubated ON at 35°C. On the next day, all Gram-negative strains detected were identified to the species level by MALDI-TOF MS (Bruker Daltonics, Bremen, Germany) and all *K*. *pneumoniae* isolates (n = 24) were subjected to typing by *Xba*I-PFGE as described [28,38]. PFGE profiles were compared with the *Xba*I-PFGE profile of strain CTX-M-15-Kp in BioNumerics version 6.6 (Applied Maths NV, St-Martens-Latem, Belgium) and interpreted according to the Tenover criteria [39].

***In vitro*:** The segregational stability of the CTX-M-15-encoding plasmid in CTX-M-15-Kp was also investigated by serial passage *in vitro*. Two independent populations of each ancestor isolate (the first and last CTX-M-15-Kp isolate from child 1–4, Table 3) were propagated in 1 mL LB broth in 96 x 2 ml deepwell plates at 37°C with shaking at 160 rpm (Vibrax orbital shaker; IKA, Staufen, Germany). Plate lids with 1.5 mm holes were used to prevent evaporation. We used a checker-board pattern to arrange the populations in the plates. In this set-up the four nearest wells to each population contained uninoculated medium. This allowed prevention and monitoring of cross-contamination between evolving populations as described [40]. Twice a day for ~two months 10 μL were serially transferred into 1 mL of fresh medium, allowing ~6.64 generations per transfer. At the endpoint of the experiment, 150 μl of 10^-5^-dilutions from the 16 evolved populations were plated on plain LB plates and incubated at room temperature ON. To determine the rate of plasmid-free segregants (i.e. CTX-M-15-Kp colonies devoid of the CTX-M-15 phenotype) in each sample, 120 colonies were randomly picked from each plate and streaked on plain LB plates and on LB plates supplemented with cefotaxime (8 mg/L) in parallel. Putative segregants were further analysed by relevant gradient tests (Liofilchem, Roseto degli Abruzzi, Italy), *bla*_CTX-M_ PCR and *Xba*I-PFGE as previously described [28,38], and by *S1*-nuclease PFGE and PBRT as described above.

**Table 3:** Segregant frequencies in evolved CTX-M-15-Kp populations and fitness estimates of plasmid-free segregant isolates.

Subject	Ancestor isolate		Segregant frequency^a^		Relative fitness of segregant isolates^b^ *w* (95% CI)	*P* value^c^	Relative growth rates of segregant isolates^d^ (95% CI)	*P* value^e^
	Sample	ID	Pop 1%	Pop 2%				
Child 1	First	Kp2158	0.83	4.17	1.02 (0.93–1.12)	0.615	1.027 (1.007–1.047)	0.01*
	Last	Kp734	1.67	2.50	0.97 (0.92–1.02)	0.203	0.950 (0.926–0.975)	<0.001*
Child 2	First	Kp2180	12.50	5.00	0.96 (0.92–0.99)	0.02*	1.017 (0.992–1.045)	0.189
	Last	Kp723	0.83	6.67	0.97 (0.91–1.02)	0.208	1.004 (0.978–1.031)	0.751
Child 3	First	Kp2223	3.33	1.67	0.98 (0.95–1.02)	0.309	1.023 (0.997–1.050)	0.078
	Last	Kp798	17.50	6.67	0.97 (0.93–1.02)	0.201	1.003 (0.964–1.039)	0.863
Child 4	First	Kp2166	4.17	13.85	0.99 (0.93–1.05)	0.613	1.013 (0.997–1.031)	0.138
	Last	Kp848	4.17	5.38	1.01 (0.96–1.06)	0.721	1.030 (0.999–1.056)	0.039*

^a^Endpoint segregant frequency after serial transfer for ~ 430 generations (two evolved populations, Pop1 and Pop 2, per ancestor isolate).

^b^The ratio of the Malthusian parameters of the plasmid-free segregant to that of its plasmid-carrying evolved ancestor isolate. A relative fitness of 1.0 denotes no difference in relative fitness (i.e. no cost of plasmid carriage). A relative fitness >1.0 is indicative for plasmid-carriage associated fitness costs, whereas values <1.0 are indicative for a benefit of plasmid-carriage to the host strain. Number of parallels: n = 9

^c^Standard one-sample *t*-test, two tailed.

^d^The ratio of the growth rate of the plasmid-free segregant to that of its plasmid-carrying evolved ancestor isolate. Relative growth rates >1.0 are indicative for plasmid-carriage associated fitness costs, whereas values <1.0 are indicative for a benefit of plasmid-carriage to the host strain. Number of parallels: n = 9, except Kp2158 n = 7.

^e^Standard two-sample *t*-test, two tailed.

*Significant values (*P<*0.05).

### Biological cost of plasmid carriage in CTX-M-15-Kp

The biological cost associated with carriage of the CTX-M-15-encoding plasmid was estimated for the eight evolved CTX-M-15-Kp plasmid-carrying isolates (ancestral isolates, Table 3) and eight corresponding plasmid-free segregant isolates in two ways: (i) pairwise head-to-head competitions (mixed culture competitions) between evolved plasmid-carrying ancestral isolates and their corresponding plasmid-free segregant isolates, and (ii) comparison of growth rates of these populations in monoculture as a proxy for biological fitness.

**Culture conditions:** All incubations were carried out at 37°C. Bacteria were grown in LB broth with shaking at 160 rpm or on LB plates. From freeze cultures of evolved CTX-M-15-Kp plasmid-carrying isolates and their corresponding plasmid-free segregant isolates, all recovered at the endpoint of the serial passage experiment described above, cells were streaked on LB plates (with cefotaxime 8 mg/L and without cefotaxime, respectively) and incubated ON. From these plates, single colonies were picked, inoculated in 2 mL LB broth and incubated ON. The ON cultures were serially diluted 1:1000 in a 2 mL culture volume and incubated for another 14 h before fitness experiments were conducted.

**Pairwise competitions:** Competition experiments were performed in triplicates and repeated three times as previously described [41,42]. The competitors were mixed 1:1 (~ 2.5x10^6^ cells of each) in 1 mL LB broth and incubated for 12 h, allowing 8–9 generations per competition. Initial (N_0_) and final densities (N_12_) of each competitor (cfu/mL) were determined by selective (cefotaxime 8 mg/L) and non-selective plating. Relative fitness (*w*) [41] was estimated as described previously [42]. Briefly, the population growth of each competitor, known as its Malthusian parameter (*m*) [41], was determined using the equation *m* = ln(N_12_/N_0_). The value *w* for each segregant isolate was estimated as a ratio of the Malthusian parameter of the plasmid-free segregant isolate (*m*_1_) to that of its plasmid-carrying ancestral isolate (*m*_2_) [41].

**Growth rates:** Growth rates of plasmid-carrying isolates and their corresponding plasmid-free segregant isolates were determined during logarithmic growth using the VersaMax Microplate Reader (Molecular Devices, Sunnyvale, USA). Approximately 2x10^5^ cells of each population were inoculated into 200 μL LB broth and incubated with shaking in monocultures. Each isolate was assayed in three independent cultures with three technical replicates per culture in at least two independent experiments. Absorbance was recorded at 650 nm at regular intervals of five minutes. Growth rate calculations were based on OD_650 nm_ values between 0.1 and 0.6, where growth was observed to be exponential using the program GrowthRates [43]. Every growth rate calculation was manually inspected for trustworthiness. Relative growth rates were calculated as the growth rate of a plasmid-free segregant isolate divided by the growth rate of the corresponding plasmid-carrying ancestral isolate.

**Statistics:** Relative growth rates and *w*-values are reported with 95% confidence intervals (CI). Statistical analysis was performed using Student’s *t*-test (two-tailed, independent samples). The significance level was set to 95% (*P<*0.05).

## Results

### Maintenance of a *bla*_CTX-M-15_-bearing IncFII_K_ plasmid in CTX-M-15-Kp during two years of intestinal colonization

**Fig 1:**
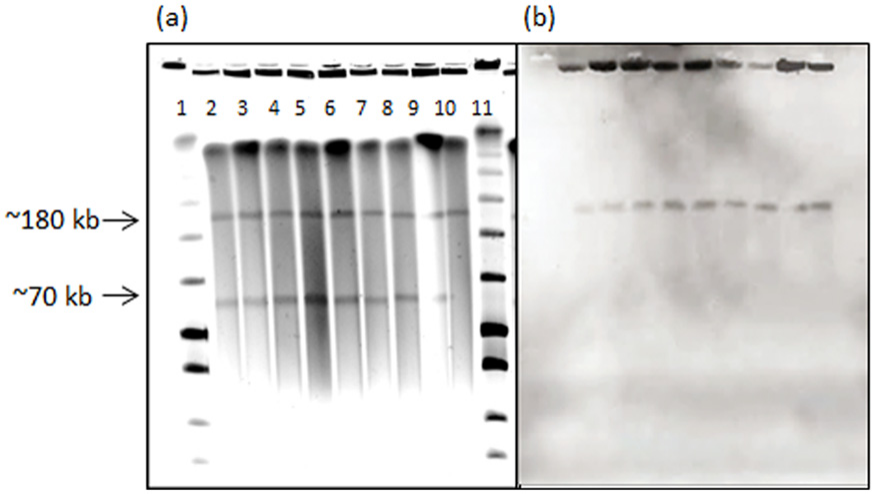
Plasmid profiles of nine CTX-M-15-Kp isolates. (a) PFGE profiles of *S1*-nuclease digested total DNA of selected CTX-M-15-Kp isolates, including the initial breast milk isolate from the mother of child 1 (index isolate), and the first and the last faecal isolates obtained from the four children colonized the longest (child 1–4). Lane 1 and 11: Low Range ladder; Lane 2: Breast milk isolate (Kp2177); Lane 3: Child 1 first (Kp2158); Lane 4: Child 2 first (Kp2180); Lane 5: Child 3 first (Kp2223); Lane 6: Child 4 first (Kp2166); Lane 7: Child 1 last (Kp734); Lane 8: Child 2 last (Kp723); Lane 9: Child 3 last (Kp798); Lane 10: Child 4 last (Kp848). (b) Southern blot-hybridization of *S1*-nuclease digested DNA using a *bla*_CTX-M_ specific probe.

Nine of 10 CTX-M-15-Kp isolates carried a ~70 kb and a ~180 kb plasmid and were PCR-positive for both the IncR and IncFII_K_ replicons. The plasmid profiles of all included CTX-M-15-Kp isolates, except Kp2189 from child 5, are shown in Fig. 1a. The follow-up isolate from child 4 (Kp848) carried only the ~180 kb plasmid (Fig. 1a) and was positive only for the IncFII_K_ replicon. Southern blot-hybridization results confirmed the location of IncR on the ~70 kb plasmid, and both *bla*_CTX-M_ and IncFII_K_ on the ~180 kb plasmid. Results are summarized in Table 1. Stable *S1-*nuclease PFGE-profiles from the first to the last isolates of child 1–4 (Fig. 1a), suggest that the ~180 kb *bla*_CTX-M-15_-bearing plasmid (Fig. 1b) persisted in CTX-M-15-Kp during the entire colonization period of up to two years.

None of the 24 ESBL-negative *K*. *pneumoniae* isolates recovered from ESBL-negative follow-up samples from 35 children were clonally related to strain CTX-M-15-Kp when investigated by *Xba*I-PFGE, supporting the hypothesis that loss of *bla*_CTX-M-15_ in the children was due to elimination of strain CTX-M-15-Kp rather than segregational plasmid loss or loss of *bla*_CTX-M-15_.

### Plasmid pKp848CTX encodes multiple antibiotic-, heavy metal- and thermoresistance determinants

The CTX-M-15-encoding plasmid isolated from Kp848 (the last follow-up isolate from child 4) was named pKp848CTX. The complete plasmid sequence consisted of 182 204 bps, with an average GC content of 51.6%. In total 185 ORFs were predicted and annotated for plasmid pKp848CTX (GenBank accession number LM994717). An IncFII_K_ replicon was identified on pKp848CTX and further typed to IncFII_K2_.

**Fig 2:**
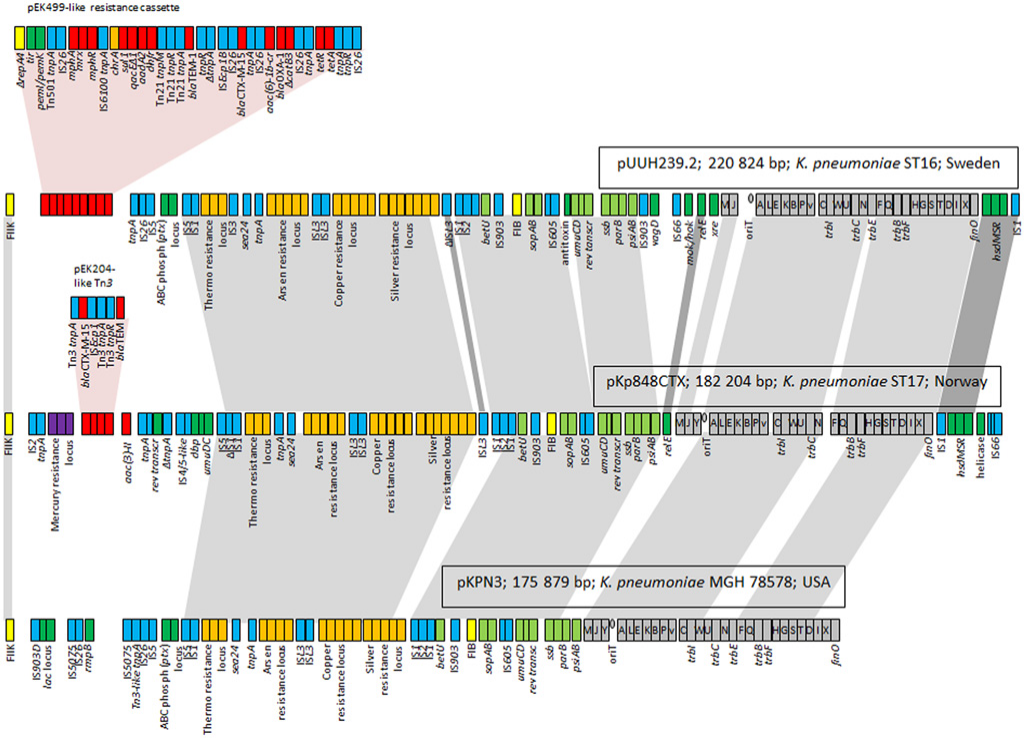
Major structural features of pKp848CTX compared with pKPN3 and pUUH239.2. Yellow boxes indicate replicase genes, grey boxes indicate the transfer region, light green boxes indicate core genes common in the three plasmids, dark green boxes indicate core genes not common in all three plasmids, orange boxes indicate heavy metal- and thermoresistance gene clusters, purple boxes indicate mercury resistance gene cluster, red boxes indicate antibiotic resistance genes and blue boxes indicate IS and transposon related genes. Light grey shadings indicate regions common for all three plasmids. Dark grey shadings indicate regions common among two of the plasmids.

The backbone of pKp848CTX consisted of a conserved transfer region and several gene clusters involved in plasmid survival and maintenance. The pKp848CTX backbone revealed high sequence similarity to pKPN3 and other pKPN3-like plasmids [15,17,19,20,23] (Fig. 2); including the Swedish pUUH239.2, isolated from a CTX-M-15-producing *K*. *pneumoniae* ST16 outbreak strain [20], and the Czech pKPN_CZ, isolated from a CTX-M-15-producing *K*. *pneumoniae* ST416 outbreak strain [23].

Plasmid pKp848CTX carried the antibiotic resistance genes *bla*_CTX-M-15_ (linked to IS*Ecp1*) and *bla*_TEM-208_ within a Tn*3*-like transposon. The Tn*3-*coupled antibiotic resistance region of pKp848CTX was similar to that described in pEK204 and pEK516, isolated from *E*. *coli* ST131 strains in the UK [44]. Furthermore, the aminoglycocide resistance determinant *aac(3)-IIa* was located downstream, and a mercury resistance gene cluster was located upstream of the Tn*3*-like transposon. A second variable region encoded several gene clusters involved in heavy metal- (copper, silver and arsenic) and thermoresistance. The heavy metal- and thermoresistance encoding regions in pKp848CTX are highly similar to corresponding regions in pKPN3 (GenBank accession number CP000648) and pUUH239.2 (GenBank accession number CP002474) (Fig. 2).

### No evidence for self-transfer of pKp848CTX

***In vivo*:** During more than two years of follow-up of 49 children colonized with strain CTX-M-15-Kp [29], screening of 589 faecal samples obtained from these children for CTX-M-15-producing Enterobacteriaceae other than CTX-M-15-Kp returned only a single CTX-M-15-producing *E*. *coli* isolate (Ecol567). Ecol567 carried plasmids of different sizes and other replicon types than CTX-M-15-Kp, and *bla*_CTX-M-15_ was confirmed on a ~75 kb plasmid in this isolate (Child 5, Table 1). Thus, we have no evidence of horizontal transfer of pKp848CTX from CTX-M-15-Kp to other Enterobacteriaceae during the intestinal colonization period in these 49 children.

***In vitro*:** Plasmid pKp848CTX could not be transferred from CTX-M-15-Kp to *E*. *coli* J53–2 (detection limit: ~1x10^-9^/donor), or re-transferred to isogenic plasmid-free *K*. *pneumoniae* segregant isolates (~3.3x10^-9^/donor), by broth- or filter mating as described above. The finding that pKp848CTX was not re-transferred into the genetic background it originated from, supports our *in vivo* results and significantly strengthens the hypothesis that pKp848 is not self-transferable when hosted by CTX-M-15-Kp.

### Segregational loss of pKp848CTX is highly variable *in vitro*

The endpoint frequency of putative segregants after ~430 generations of serial passage (including 16 CTX-M-15-Kp populations) ranged from 0.83% to 17.5% (Table 3). There was no systematic difference in segregant frequencies between the first and the last isolates from individual children, or between isolates from different children. Segregational loss of the CTX-M-15-encoding plasmid was confirmed by phenotypic and molecular analyses in at least one putative segregant isolate from each of the eight evolved ancestor isolates. These results contrast our *in vivo* results, where pKp848CTX-like plasmids were stably maintained by CTX-M-15-Kp during two years of intestinal colonization in four children.

### Proxies for fitness reveal neutrality of pKp848CTX carriage in CTX-M-15-Kp

**Pairwise competitions:** Estimates of relative fitness (*w*) of plasmid-free segregant isolates were determined in pairwise competitions between segregant isolates and their corresponding plasmid-carrying CTX-M-15-Kp ancestral isolates during 12 hours including lag, log, and stationary phase. We found that plasmid pKp848CTX appeared adapted to its host. Differences in relative fitness from neutrality (*w* = 1.0) varied within an interval of 6% (*w* = 0.96 (Kp2180) to *w* = 1.02 (Kp2158), Table 3), and revealed a statistical significant reduction of fitness in only one segregant isolate (Kp2180, *w* = 0.96, *P* = 0.02, Table 3) suggesting a subtle benefit of plasmid carriage under the *in vitro* experimental conditions. In accord with a low to neutral cost scenario, no difference in fitness was found between the first and the last isolate of individual children (all *P*>0.05, *t*-tests), indicating that plasmid carriage costs did not change within the two years of human colonization in four children. These results furthermore suggest that the variability in segregant frequencies observed between isolates was probably not related to fitness costs of plasmid carriage.

**Growth rates:** A fitness estimate derived from pairwise competition experiments can be influenced by marker effects on selective media. Although no such effects were observed, we sought to confirm the low impact of pKp848CTX on host fitness by measuring the maximal growth rates of plasmid-carrying and corresponding plasmid-free segregant isolates, as an alternative proxy for fitness. Generation times of the CTX-M-15-Kp isolates, with and without pKp848CTX, varied between 30.24 and 33.14 min (average 31.17 ± 0.66). When growth rates of segregant isolates were expressed relative to the ancestral isolates, we found that two plasmid-free segregant isolates (Kp2158 and Kp848) displayed statistically significant increased growth rates indicating a biological cost of plasmid carriage (Table 3). One segregant (Kp734) displayed a statistically significant reduced growth rate (Table 3) suggesting a small benefit of plasmid carriage after two years of colonization. However, these results were not consistent with the results from the head-to-head competition experiments and should be interpreted with caution.

## Discussion

Plasmid stability, plasmid-host adaptation and the level of plasmid-conferred fitness costs are of importance for the persistence and spread of antibiotic resistance determinants. Here, we show that a ~180 kb IncFII_K_ plasmid (pKp848CTX) persisted to carry *bla*_CTX-M-15_ and was stably maintained by a *K*. *pneumoniae* ST17 strain (CTX-M-15-Kp), with low costs to the bacterial host fitness, during intestinal carriage in four children for up to two years. Moreover, the DNA sequence of pKp848CTX uncovered several genetic elements, encoding phenotypic traits, which may have contributed to increased survival and persistence of CTX-M-15-Kp in the hospital environment during the NICU outbreak.

The CTX-M-15-producing *K*. *pneumoniae* ST17 strain caused a NICU outbreak in our hospital during 2008–09 [28]. ST17 is a single locus variant of ST16. CTX-M-15-producing *K*. *pneumoniae* ST16 caused a large hospital outbreak at Uppsala University Hospital in Sweden in 2005 [22] and has been reported as one of the most prevalent lineages, along with ST15, to have established itself in the Copenhagen area [45], indicating that strains belonging to this lineage might be common in Scandinavia. Moreover, *K*. *pneumoniae* ST17 producing CTX-M-15 has been reported from different countries and in different settings; from wastewater treatment plants in Algeria to clinical isolates in Canada and strains causing infection or colonization in newborn Spanish children [46–48].

To our knowledge, the IncFII_K_ plasmid has not yet been described to carry *bla*_CTX-M-15_ in *K*. *pneumoniae* ST17, but in *K*. *pneumoniae* of ST16, ST416, ST15, ST48 and ST23 [19,20,23]. IncFII_K_ plasmids are reported to carry *bla*_KPC-2 and 3_ in *K*. *pneumoniae* ST258 [15,17]. The worldwide dissemination of pKPN3-like plasmids into diverse *K*. *pneumoniae* strains indicates high stability and robustness of this backbone. Plasmid pKp848CTX seem to have evolved by the transposition of a Tn*3*-like transposon into a pKPN3-like backbone, whereas the related pUUH239.2 evolved by IS*26*-mediated integration of a completely different antibiotic resistance cassette into a similar backbone [20].

Plasmid pKp848CTX carries multiple antibiotic-, heavy metal- and thermoresistance determinants, features which may have favoured CTX-M-15-Kp in the hospital environment. The thermo-, arsenic-, copper- and silver resistance cluster in pKp848CTX is flanked by IS-elements, potentially representing a composite transposon (Fig. 2). Similar gene clusters seem to be conserved in other IncFII_K_ pKPN3-like outbreak plasmids, such as pUUH239.2 and pKPN_CZ [20,23]. The thermoresistance gene cluster in pKp848CTX encodes a Clp ATPase highly similar to ClpK, recently described in thermotolerant *K*. *pneumoniae* strains by Bojer *et al*. [49]. We did not investigate the phenotype of CTX-M-15-Kp with respect to heat resistance or tolerance, but a thermoresistant phenotype would allow a bacterial strain to survive common disinfection procedures and increase its survival in the hospital environment and on medical devices [23,50]. Thus, thermoresistance coupled with multiple heavy metal- and antibiotic resistance determinants, could to some extent explain why CTX-M-15-Kp survived and spread so efficiently in the NICU-environment and among infants during the outbreak [28].

We did not identify any known virulence determinants or colonization traits on plasmid pKp848CTX (Fig. 2). In previous studies, two isolates of strain CTX-M-15-Kp ST17 (the breast milk index isolate and a blood culture isolate from one of the colonized children) were screened for genes encoding common virulence factors in *K*. *pneumoniae* (*rmpA*, *wcaG*, *allS* and genes encoding capsule types K1, K2, K5, K20, K54, K57), but none of these were detected [28]. However, undetected colonization or virulence factor(s) may have played a role in persistence of pKp848CTX. Currently, several isolates of strain CTX-M-15-Kp are being sequenced to explore the characteristics of this ST17 strain, and future results may give us new insight into chromosomal traits relevant for the virulence or colonization abilities of this strain.

Our attempts in transferring pKp848CTX to *E*. *coli* and back into segregant CTX-M-15-Kp genetic backgrounds *in vitro*, remained unsuccessful. We did also not obtaine evidence in favour of horizontal spread of pKp848CTX *in vivo* by screening all follow-up samples from 49 colonized children for CTX-M-15-producing Enterobacteriaceae other than CTX-M-15-Kp_._ Taken together, these data suggest that pKp848CTX does not spread easily to other hosts by conjugation, which may have limited the dissemination and persistence of the plasmid and its resistance traits both in the hospital environment and in the intestine of colonized children. However, unexplored experimental conditions as well as recipient genetic backgrounds could have favoured successful *in vitro* transfer of pKp848CTX.

Plasmid pKp848CTX carries a conserved transfer region similar to the transfer regions of the conjugative IncFII_K_ plasmids pUHH239.2 and pKPN_CZ. Both pUUH239.2 and pKPN_CZ were shown to be transferable to *E*. *coli in vitro*, and pUUH239.2-like and pKPN_CZ-like plasmids were detected in other clinical Enterobacteriaceae strains during the respective hospital outbreaks indicating *in vivo* transfer of these plasmids [20,21,23]. In contrast to pUHH239.2 and pKPN_CZ, pKp848CTX does not seem to be self-transferable, at least not when hosted by CTX-M-15-Kp ST17. Unlike pUHH239.2 and pKPN_CZ, pKp848CTX lacks *trbE* in its transfer region. TrbE is a member of the VirB4 type IV secretion system, and reported to be essential for the conjugation abilities of plasmid RP4 in *E*. *coli* [51]. The significance of this finding is unknown, but the absence of *trbE* might be one explanation for the observed restricted self-transfer abilities of pKp848CTX. One could also speculate if the impaired self-transferability of pKp848CTX when hosted by CTX-M-15-Kp is due to a successful plasmid-host adaptation, and that pKp848CTX would in fact be self-transferable if transferred into a new host. It was, however, not within the scope of this study to examine the properties of pKp848CTX when hosted by other strains.

Supported by stable *S1*-nuclease profiles, PBRT and Southern blot results, we suggest that pKp848CTX-like plasmids were maintained by strain CTX-M-15-Kp throughout two years of intestinal colonization in four young children, who did not receive antibiotics during follow-up. Furthermore, we found no evidence of plasmid loss during intestinal carriage of CTX-M-15-Kp in 35 children as we screened their two first ESBL-negative follow-up samples for isogenic plasmid-free *K*. *pneumoniae* isolates. These results may also suggest that plasmid pKp848CTX was well adapted to CTX-M-15-Kp and conferred negligible fitness cost to its host *in vivo*, and that the elimination of CTX-M-15-Kp colonization in the children was due to outnumbering of the strain per se rather than plasmid loss.

pKp848CTX encodes several post-segregational killing systems (Fig. 2) likely to prevent segregant formation through improper plasmid partitioning upon cell division. The question is how functional or well-tuned these systems really are. In contrast to Sandegren *et al*., who reported no segregational loss of pUUH239.2 in *K*. *pneumoniae* over 1250 generations *in vitro* [20], plasmid-free segregants appeared at frequencies ranging from 0.83% to 17.5% after ~430 generations, without antibiotic selection, in our serial passage experiment. The reason for this striking and surprisingly variable pattern may be owed to our 10-fold decreased bottleneck, as compared to Sandegren *et al*. [20], which may have reduced the probability for stochastic loss of initially rare segregants from some of the populations. Host-strain specific traits involved in plasmid stability can also not be ruled out. However, variability in segregant frequencies observed in this study, is likely to be representative for evolving population where segregants emerge stochastically and escape bottlenecks at different time points, and thus rise in frequency at various time points resulting in diverse endpoint frequencies. Our inability to detect transconjugants in mating experiments between CTX-M-15-Kp and plasmid-free segregant isolates of CTX-M-15-Kp, does not rule out that transfer could have occurred beyond our limit of detection. Unrecognized transfer could have altered plasmid loss rates to an unknown extent.

The appearance of segregants in antibiotic free serial passage cultures suggested that pKp848CTX carriage was associated with a reduction in host competitive fitness. However, head-to-head competition experiments did not reveal such a cost under the experimental conditions applied. Estimates of relative growth rates suggested that one segregant strain suffered slightly reduced maximum growth rates, a result indicative of a beneficial plasmid-host association, as previously reported [24,27]. On the other hand, two segregants displayed increased growth rates indicating that pKp848CTX reduce fitness of its host. However, the growth rate results should be interpreted with caution since they merely reflect a short time-span of logarithmic growth, whereas the mixed culture competition experiments include the complete growth cycle of the two competitors. Taken together, the presented data suggest low, if any, biological cost of pKp848CTX carriage.

Although we did not assess plasmid loss rates at different time points during the serial passage experiment, we suggest that the relative instability of pKp848CTX reported here is not due to plasmid-mediated reduced host fitness. Our results are consistent with de Gelder and co-workers, who suggested that initial changes in the ratios of segregants versus plasmid-carrying cells are dominated by segregational loss rates, whereas after enrichment of plasmid-free cells, the overall greater fitness of segregants would allow them to prevail [52]. We argue that this is particularly the case when plasmids impose low metabolic burden on their hosts and we further stress that our experimental approach cannot detect differences in relative fitness below 1% [41]. Hence, as assumed [52], the trajectory of the rise of segregants in populations during evolution in a non-selective environment is probably still driven by alleviation of fitness cost after plasmid loss, and not by constant influx of newly emerging segregants.

In conclusion, pKp848CTX encodes multiple antibiotic-, heavy metal- and thermoresistance determinants, which may have contributed to the fitness and survival of CTX-M-15-Kp in the hospital environment during the NICU outbreak. Plasmid pKp848CTX was stably maintained in its *K*. *pneumoniae* ST17 host throughout human intestinal colonization for two years, conferring negligible fitness cost to its host. Our data suggest that pKp848CTX may have been well adapted to its host before it appeared in the NICU and before colonizing affected children.
